# The impact of Ricinus straw on tomato growth and soil microbial community

**DOI:** 10.3389/fmicb.2024.1499302

**Published:** 2024-12-02

**Authors:** Jingyu Zhang, Minghao Liu, N’da Brou Jean Landry, Yaping Duan, Xin Li, Xingang Zhou

**Affiliations:** ^1^Key Laboratory of Biology and Genetic Improvement of Horticultural Crops (Northeast Region), Ministry of Agriculture and Rural Affairs, Northeast Agricultural University, Harbin, China; ^2^Department of Horticulture, Northeast Agricultural University, Harbin, China; ^3^School for the Engineering of Matter, Transport and Energy, Arizona State University, Tempe, AZ, United States

**Keywords:** Ricinus straw, tomato growth, bacterial community, fungal community, *Fusarium* sp.

## Abstract

Returning straw can alter the soil microbial community, reduce the occurrence of soilborne diseases, and promote plant growth. In this study, we aimed to evaluate the effects of Ricinus straw on tomato growth and rhizosphere microbial community. We carried out microcosm experiments to investigate the effects of Ricinus straw with different dosages (0, 1, and 3%) on tomato dry biomass and rhizosphere bacterial and fungal communities. The results indicated that the dry biomass of tomato seedlings with 1% addition of Ricinus straw increased by 53.98%. In addition, Ricinus straw also changed the abundance, diversities, and composition of tomato rhizosphere microbial communities. In detail, the addition of 1% Ricinus straw increased the relative abundance of putative beneficial bacteria and fungi in straw decomposition, such as *Ramlibacter* sp., *Azohydromonas* sp., *Schizothecium* sp., and *Acaulium* sp., and decreased the relative abundance of *Fusarium* sp. Meanwhile, Ricinus straw inhibited the growth of putative pathogenic microorganisms. The correlation analysis showed that the changes in fungal community operational taxonomic units stimulated by the addition of Ricinus straw may play a crucial positive regulatory role in tomato growth. Finally, the representative fungal strain *Fusarium oxysporum f.* sp. *Lycopersici* (FOL), named TF25, was isolated and cultured. We found that Ricinus straw extract inhibited the growth of TF25 in an *in vitro* experiment with an inhibition rate of 34.95–51.91%. Collectively, Ricinus straw promoted plant growth by changing the rhizosphere microbial community composition and inhibiting FOL growth, which provides new evidence for understanding the improvement of key microorganism composition in improving crop growth and the sustainability of agriculture.

## 1 Introduction

The large-scale application of chemical fertilizers significantly elevates crop yield. At the same time, the excessive and prolonged use of fertilizers may lead to a decline in soil organic matter content when the amount of C input from external sources is less than what is required for microbial decomposition (Guo et al., 2010; Jiang et al., 2014). Therefore, improving the content of soil organic carbon plays a significant role in enhancing soil fertility and maintaining soil productivity (He et al., 2015; Su et al., 2020). Straw, as an important biological resource, can release polysaccharides, fats, and other intermediate products during the decomposition process, ultimately forming soil organic matter, further enriching the soil carbon pool, and altering soil physical properties (Abdulla and El-Shatoury, 2007; Schnitzer and Monreal, 2011). In addition, returning straw has positive effects on improving soil structure, water holding capacity, nutrient availability, microecological environment, and increasing crop yield (Lenka and Lal, 2013; Xu et al., 2019; Yang et al., 2021).

The decomposition process of the straw can release mineral nutrients that are easily absorbed and utilized by crops, which is beneficial to crop growth and productivity while also reducing environmental pollution (Zhang et al., 2017). For instance, in the semi-arid regions of Northwest China, applying 1% of wheat straw back into the soil improved the physical and chemical properties of the soil, promoting the growth of tomato seedlings (Khan et al., 2019). In addition, returning straw not only increases soil moisture content and promotes nutrient cycling but also has a positive regulatory effect on the growth of sunflower roots (Chen et al., 2024). Moreover, the small molecular organic compounds such as amino acids, organic acids, and peptides released during straw decomposition can also stimulate root development. Meanwhile, studies have found that ground straw exhibits a higher decomposition rate, which is beneficial for the dry matter accumulation of crops such as rice and corn (Zhao et al., 2021; Lv et al., 2024).

Soil microorganisms, as an essential component of the soil ecosystem, are vital factors influencing soil nutrient cycling and plant health and productivity in both natural and agricultural ecosystems (Hollister et al., 2013). The composition of soil microbial communities influences soil microbial community functions, and conversely, the composition of soil microbial communities also changes with variations in soil microbial functions (Bever et al., 2012). Some studies have confirmed the impact of straw decomposition on microbial community abundance and diversity, with a significant increase in soil microbial biomass and activity observed after returning straw (Marschner et al., 2011; Xia et al., 2020; Deng et al., 2021). Meanwhile, some scholars have researched the optimal amount of straw return to the field. Different amounts of straw return can induce changes in soil microbial communities, and such variations may alter the functionality of soil microbial communities. However, the majority of studies have only focused on the response of soil microbial communities to different crop straw types and amounts of return to the field (Almagro et al., 2021; Zhang et al., 2024), and further clarification is needed on how these microbial community changes affect functionality and influence crop growth.

Crop straw contains nutrients such as nitrogen, phosphorus, and potassium and organic compounds such as cellulose, hemicellulose, lignin, proteins, and carbohydrates. The diversity of its material composition determines the diversity of microorganisms. The functionality of soil microorganisms is usually not determined by a single species, but rather by the richness and diversity of the community, which are key influencing factors for microbial functions. Several studies have found that bacteria, fungi, and actinomycetes decompose cellulose, hemicellulose, and lignin by secreting a series of cellulolytic enzymes (Liang et al., 2015). As straw decomposes, different microbial communities exhibit variations in using straw components (Wagg et al., 2019). Among them, copiotrophic bacteria such as Proteobacteria and Bacteroidetes dominate straw decomposition in the early stages. In this process, bacteria mainly decompose soluble sugars, starch, cellulose, and hemicellulose in the straw (Marschner et al., 2011; Surey et al., 2020). Firmicutes and the majority of fungal communities (Ascomycota and Basidiomycota) play a vital role in the later stages of straw decomposition, leading to the decomposition of recalcitrant hydrophobic organic compounds (lignocellulose and lignin) (Cotrufo et al., 2015; Zhang et al., 2021). In conclusion, the return of straw to the field plays a crucial role in regulating the structure and composition of soil microbial communities.

Based on the findings of the above studies, the return of straw can promote crop growth, and alter microbial community structure. Field crops, such as wheat, corn, and rice, have been widely studied (Jin et al., 2019; Duan et al., 2023; Liu et al., 2023). However, the rhizospheric microbial mechanisms through which medicinal plant straw promotes tomato growth are largely unknown. Ricinus, a tropical plant of the family Euphorbiaceae, possesses anticancer, antidiabetic, antioxidant, and anti-inflammatory activities and is widely cultivated around the world (Singh and Ambika, 2009; de Assis Junior et al., 2011; Kassaye and Shibeshi, 2023), but the application effects of its straw are still unclear. Therefore, this study evaluated the effects of Ricinus straw on the growth of tomatoes and the abundance and diversity of the rhizosphere microbial community and investigated the correlations between bacterial and fungal communities and plant growth. First, the study investigated the impact of Ricinus straw at different stages of decomposition on tomato growth. Second, the composition of rhizosphere microbial communities induced by varying levels of straw addition was analyzed. The present study found that adding an appropriate amount of Ricinus straw can increase the abundance of beneficial microbial community in the tomato rhizosphere, thereby improving tomato growth.

## 2 Materials and methods

### 2.1 The preparation of soil and Ricinus straw

The soil used in this study was collected from a conventional agricultural field at Northeast Agricultural University, Harbin, China (45°419 N, 126°379 E). Twenty black soil (C32) cores (10 cm diameter) were pooled from the upper soil layer (0–15 cm). Soils were sieved (2 mm), and large stones and plant debris were removed. The soil was sandy loam, containing organic matter, 68.37 g/kg; inorganic N (ammonium and nitrate), 82.19 mg/kg; available P, 91.58 mg/kg; available K, 122.62 mg/kg; EC (1:2.5, w/v), 0.41 mS/cm; and pH [1:2.5, w (1 g of soil)/v (2.5 mL of water)], 7.64.

When the Ricinus (*Ricinus communis* L.) were matured, their aboveground parts were air-dried to a constant weight and mechanically crushed into a powder for use in microcosm experiments; 200 g of sieved soil was filled into the microcosm. Three treatments were set up, and each treatment involved thoroughly mixing in 0 g, 2 g, and 6 g of Ricinus straws, respectively, resulting in Ricinus straw contents (w/w) of 0% (Control), 1, and 3% in each cultivation bottle, respectively, with 15 bottles for each added amount. The microcosms were placed in a dark environment at 25°C for 30 days to decompose, and the soil moisture was regulated and maintained to approximately 65% of the field capacity by adding distilled water every 2 days.

After incubation, in order to sterilize and facilitate even germination, the tomato (*Solanum lycopersicum* L.) seeds (cv. Jinfen) were soaked in water at 55°C for 30 min and then germinated in sand in a growth chamber at 25°C. After emergence, three tomato seedlings with two cotyledons were transplanted into each bottle. Three days later, tomato seedlings were thinned to one plant per bottle. All bottles were maintained in a growth chamber (28°C day/16°C night, relative humidity of 60–80%, under 200 μmol/m^2^·s light on a 16-h light/8-h dark cycle). Soil water content was adjusted every two days with distilled water to maintain the soil moisture at approximately 65% of water holding capacity. After 30 days, the tomato seedlings were harvested (with the straw decomposed for 60 days), rinsed clean with water, and dried in a 70°C oven to a constant weight to measure the plant’s dry biomass.

Following the above method, the tomato seedlings were planted in the bottles under the same conditions for 30 days (with the straw decomposed for 90 days). Then, the tomato rhizosphere soil samples were collected as previously described (Zhou et al., 2018). In detail, we gently take out the tomato plant from the pot, giving it a slight shake until a small amount of rhizosphere soil adheres to the root system. Then, we carefully brush the tomato roots with a soft-bristled brush to collect a small amount of soil, which constitutes the tomato rhizosphere soil sample. Samples from five plants replicating the individual treatment were combined to make a composite sample. Therefore, there were three composite samples for each treatment. After sieving (2-mm mesh), these fresh sampled soils were stored at −80°C for DNA extraction. The dry biomass of tomato seedlings was determined following the method described above.

### 2.2 Soil DNA extraction

Soil DNA was extracted from 0.25 g of soil using the Power Soil DNA Isolation Kit (MO BIO Laboratories, Carlsbad, CA, USA) according to the manufacturer’s instructions (Zhou and Wu, 2021). Electrophoresis was performed in a 1.2% (w/v) agarose gel stained with ethidium bromide to check the yield and quality of the extractions. Each composite soil sample was extracted in triplicate, and the extracted DNA solutions were pooled as described previously (Zhou et al., 2021).

### 2.3 Amplicon sequencing and data processing

Shanghai Meiji Biological Company completed high-throughput sequencing of soil. Primer sets of F338/R806 and ITS1F/ITS2R were used to amplify V3–V4 regions of the bacterial 16S rDNA gene and the ITS1 regions of the fungal rRNA gene, respectively (Zhou et al., 2017). The reaction conditions for the bacterial community were as follows: 95°C for 5 min, 95°C for 50 s, 62°C for 30 s, and 72°C for 10 min. The reaction conditions for the fungal community were as follows: 94°C for 5 min, 94°C for 1 min, 58°C for 1 min, and 72°C for 1 min. The PCR amplification products were identified and separated using 2% agarose gel electrophoresis, and then the PCR products were purified using the DNA gel purification kit (Takara, Beijing, China). The purified products were then subjected to high-throughput sequencing.

MiSeq sequencing data results were analyzed using the QIIME (Quantitative Insights into Microbial Ecology, Version 1.9.0) software as previously described (Bolyen et al., 2019; Zhou et al., 2023). The raw sequence reads were demultiplexed before processing with FLASH, followed by quality filtering to remove low-quality fragments. To obtain the taxonomic information corresponding to each OUT, the community species composition of each sample was then statistically analyzed at various taxonomic levels: Domain, Kingdom, Phylum, Class, Order, Family, Genus, and Species; the sequences were then assembled using FLASH software, clustered with CD-HIT (Cluster Database at High Identity with Tolerance) at a 97% similarity level; and chimeric sequences were identified and removed using USEARCH7-uparse in QIIME (Edgar, 2013). The sequences were then analyzed after rarefaction to a minimum number of sequences per sample. Bacterial and fungal sequences were aligned against the Silva132 and Unite7.2 databases, respectively, with a threshold of 0.7, and chimeric sequences were removed (Quast et al., 2013). Sequences affiliated with chloroplasts, mitochondria, and archaea were excluded, and the samples were normalized to the lowest sequence count per sample prior to statistical analysis (Quast et al., 2013).

### 2.4 Isolation of the *Fusarium* sp.

*Fusarium oxysporum* (FOL) was isolated from the rhizosphere soil samples of tomato plants cultivated in the 0% Ricinus straw addition treatment. Soil samples (1 g) were added to 9 mL of sterile water. The soil suspension was prepared using a Waring blender for 10 min. Serial dilutions from 10^−1^ to 10^−7^ cells mL^−1^ were plated on potato dextrose agar (PDA) for the cultivation of FOL. The plates were incubated for 5 days at 25°C in the dark. Five parallel concentrations were set for each soil suspension.

### 2.5 Preparation of Ricinus straw extract and potato glucose agar (PDA) medium

Based on the above finding, to verify whether Ricinus straw affected the growth of FOL, we conducted *in vitro* experiments. We evaluated the inhibitory effect of Ricinus straw extract on the growth of FOL on a PDA medium. The Ricinus straw stems were crushed and sieved through a 2-mm mesh; 105 g of Ricinus straw stems was weighed into a conical flask and added with 700 mL of distilled water, shaken at 175 rpm at a temperature of 28°C for 24 h. After shaking, the extract was filtered using cheesecloth. The filtrate was then centrifuged at 15,000 rpm for 10 min, and the supernatant was filtered through six layers of filter paper. After repeating the above steps three times, the filtrate was filtered through a 0.45-μm membrane filter and finally removed microorganisms from the filtrate through a 0.22-μm membrane filter. The potato glucose agar (PDA) medium was prepared and sterilized. At the same time, the Ricinus straw extract was preheated at 45°C in a water bath. When the temperature of the PDA medium dropped to 50°C, the extract was added to reach final concentrations of 0, 1, and 3% in the PDA medium. The colony diameter measured was determined on the 2nd, 4th, and 6th days.

### 2.6 Data analysis

Before the statistical data analysis, tests for normality and homogeneity were conducted (Levene’s test). We used SPSS (Statistical Package for the Social Sciences) software for variance statistical analysis of the data and GraphPad Prism software for bar plots. ANOVA was performed to test the effect of the addition of Ricinus straw on the dry biomasses of tomato. For more than two groups, means were compared between treatments using Tukey’s HSD test. For high-throughput data, the rarefaction curve showed that the majority of the bacterial and fungal diversity of the sample is covered by sequencing, indicating that the sequencing data of the sample are reasonable. Bacteria and fungi were compared using the Silva database and finally analyzed according to the minimum sequence number of sequencing results. QIIME was used to calculate the *α*-diversity index (Shannon index and Inverse Simpson index). The *β*-diversity was analyzed using principal coordinate analysis (PCoA) based on the Bray–Curtis distance matrix with the “Vegan” package in R language. PERMANOVA (permutations = 999) was used to analyze microbial community structure dissimilarity. A Manhattan plot was used to visualize the distribution of differential operational taxonomic units (OTUs) on the dominant bacteria and fungi with the “Edger” package in R. We conducted a correlation analysis between the OTUs with straw decomposition ability and the dry biomass of tomato plant based on Spearman’s correlation.

## 3 Results

### 3.1 Ricinus straw enhanced tomato seedling performance

**Figure 1:**
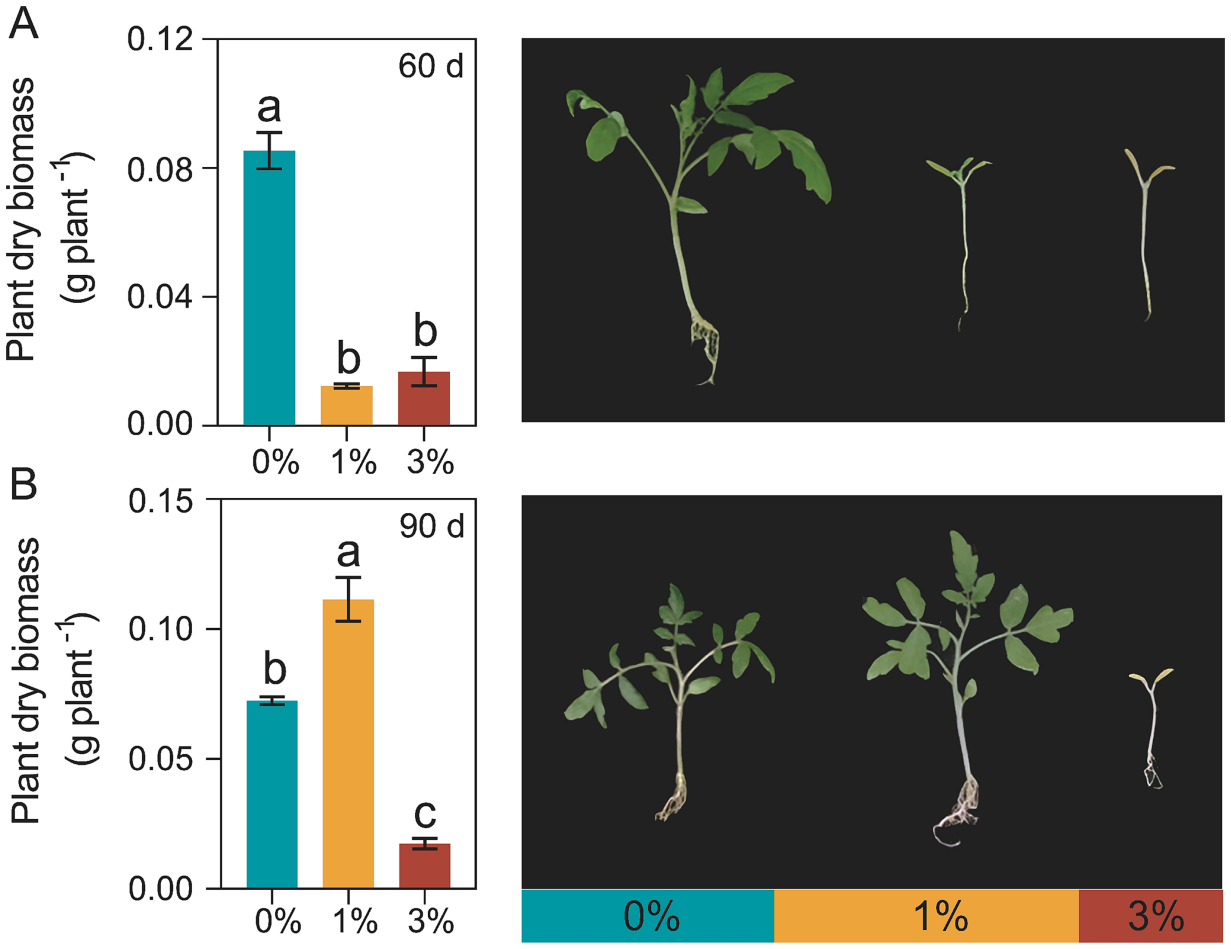
Effects of Ricinus straw on tomato seedlings dry biomass. (A) Tomato seedling dry biomass accumulation after Ricinus straw decomposition 60 days. (B) Tomato seedling dry biomass accumulation after Ricinus straw decomposition 90 days. Different letters indicate significant differences (Tukey’s HSD test, *p* < 0.05). 0, 1, and 3% represent the proportions of straw added to 200 g of soil, with no addition, 2 g, and 6 g of straw, respectively.

In the decomposition experiment (Figure 1), compared to the treatment without added straw (0%), the treatments with 1 and 3% straw addition significantly inhibited the growth of tomatoes at 60 days of decomposition, the dry biomass of tomato seedlings was decreased by 590.61 and 406.29% (Figure 1A). After 90 days of straw decomposition, the dry biomass of tomato seedlings with 1% addition was increased by 53.98 and 539.22%, compared to the 0% treatment and 3% treatment, respectively (Figure 1B).

### 3.2 Ricinus straw altered tomato rhizosphere bacterial community diversity and composition

**Figure 2:**
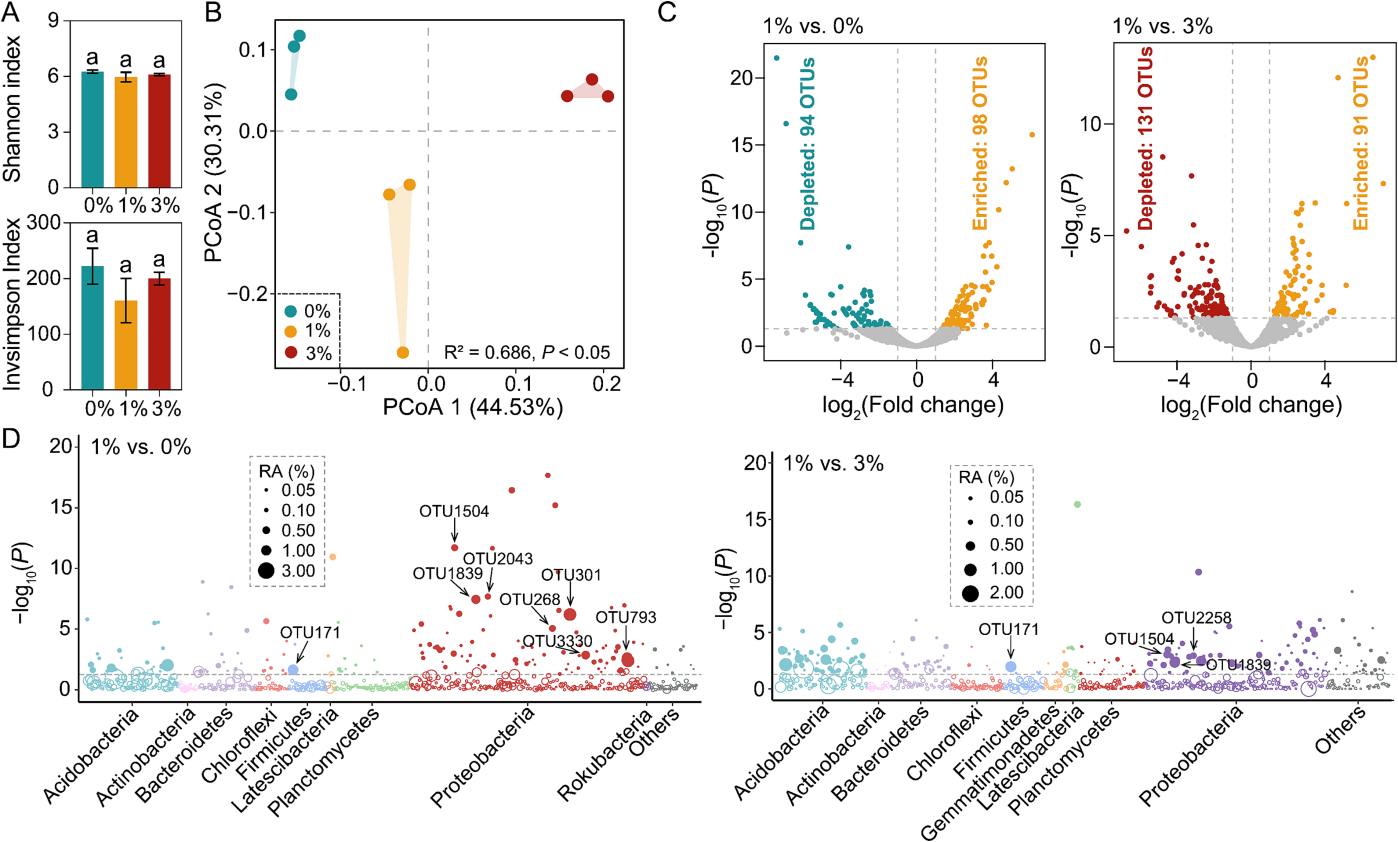
The changes of tomato rhizosphere bacterial community. (A) The *α*-diversity analysis of tomato rhizosphere bacterial community. (B) PCoA of tomato rhizosphere bacterial community *β*-diversity. (C) Volcano plot showing the enrichment or depletion of tomato rhizosphere bacterial OTUs in the 1% straw addition treatment compared to the 0 and 3% straw addition treatments, respectively (likelihood-ratio test, Benjamini–Hochberg-corrected *p* < 0.05). (D) The Manhattan plot shows the taxonomic information of OTUs enriched (*p* < 0.05, log-fold change >1) in the 1% straw addition treatment compared to the 0 and 3% straw addition treatment. The filled circles represent significantly enriched OTUs, while the open circles indicate OTUs with no significant difference. The dashed line represented the threshold of significance (Benjamini–Hochberg-corrected *p* = 0.05). 0, 1, and 3% represent the proportions of straw added to 200 g of soil, with no addition, 2 g, and 6 g of straw, respectively; OTU, operational taxonomic units.

Rarefaction curves of OTUs at 97% sequence similarity of all samples tended to approach the saturation plateau (Supplementary Figure S1). Therefore, the sequencing depth was adequate to assess the bacterial diversity of our samples. Both treatments were compared to no straw addition. First, we found that no significant difference was observed in tomato seedling rhizosphere bacterial community *α*-diversity (Figure 2A). Second, we found that based on the Bray–Curtis dissimilarities, principal coordinates analysis (PCoA) showed that bacterial communities of the addition Ricinus straw treatment differed distinctly from those of the 0%. We also found that different straw-added amounts significantly affected the bacterial communities between 1 and 3% (Figure 2B).

Across all samples analyzed, 34 bacterial phyla were detected, Proteobacteria, Acidobacteria, Bacteroidetes, Firmicutes, Actinobacteria, and Planctomycetes were the dominant phyla (average relative abundances >5%) in at least one treatment. In addition, bacterial community composition did not uniformly respond to different straw additions (Supplementary Figure S2).

The likelihood ratio test found that 98 and 94 OTUs were enriched and depleted in 1% straw addition compared to 0%, respectively; 91 and 131 OTUs were enriched and depleted compared to the 3% straw addition (Figure 2C). These stimulated OTUs mainly belonged to Proteobacteria. In particular, 1% straw addition stimulated the OTU171 (*Turicibacter* sp.), OTU1504 (*Azohydromonas* sp.), OTU1839 (*Ramlibacter* sp.) compared to the 0 and 3% straw addition (Tukey’s HSD test, *p* < 0.05). Compared to the 0% treatment, 1% straw addition stimulated the OTU268 (*Skermanella* sp.), OTU301 (*Acidibacter* sp.), OTU793 (*Lysobacter* sp.), OTU2043 (Var*iovorax* sp.), OTU3330 (*Lysobacter* sp.) (Tukey’s HSD test, *p* < 0.05). Moreover, compared to the 3% treatment, 1% straw addition stimulated the OTU2258 (*Pseudomonas* sp.) (Tukey’s HSD test, *p* < 0.05; Figure 2D), these changed species possess the functions of decomposition and promotion of plant growth.

### 3.3 Ricinus straw altered tomato rhizosphere fungal community diversity and composition

**Figure 3:**
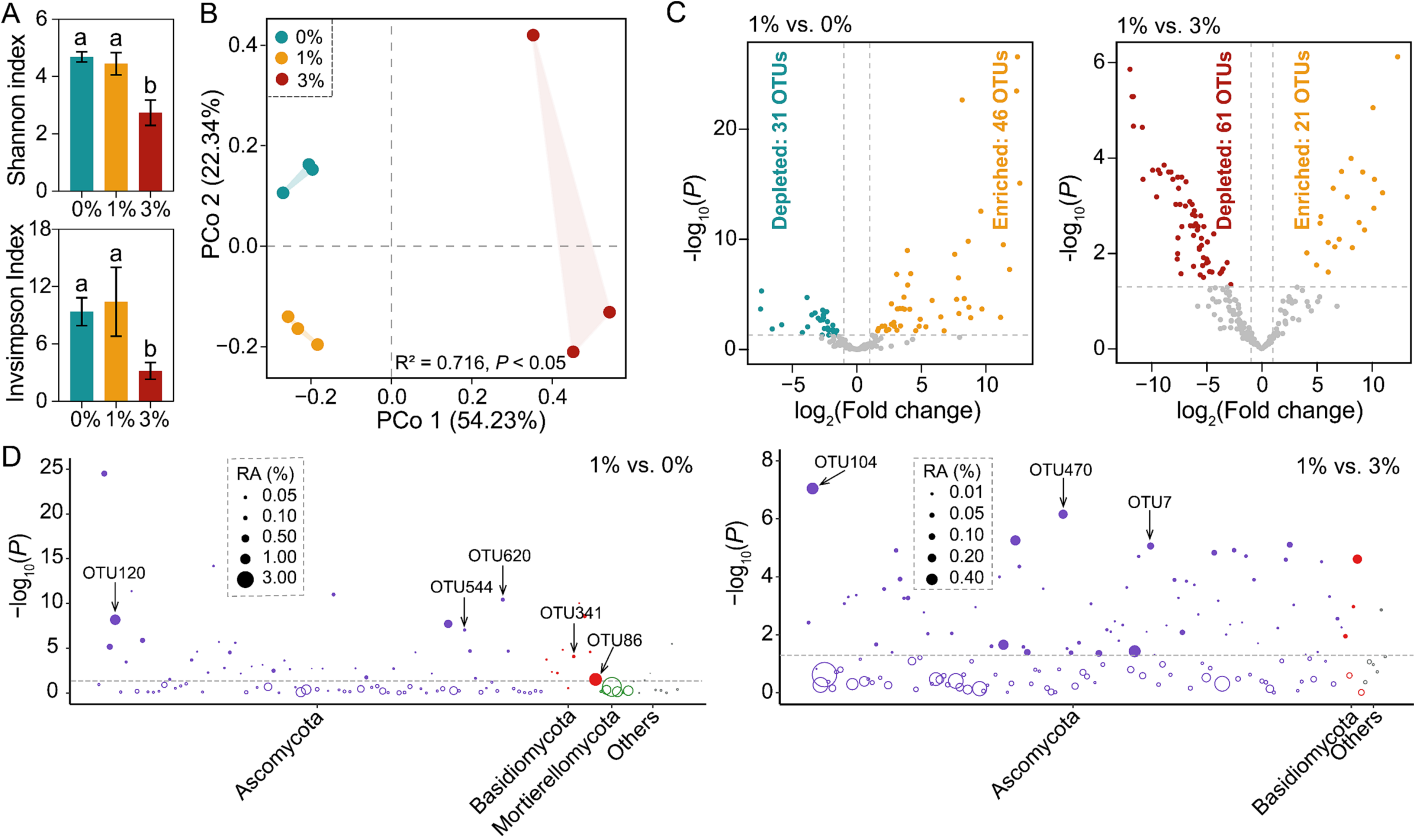
Changes of tomato rhizosphere fungal community in the decomposition experiment. (A) α-diversity analysis of tomato rhizosphere fungal community. (B) PCoA of tomato rhizosphere fungal community β-diversity. (C) Volcano plot showing the enrichment or depletion of tomato rhizosphere fungal OTUs in the 1% straw addition treatment compared to the 0 and 3% straw addition treatments, respectively. (likelihood-ratio test, Benjamini–Hochberg-corrected *p* < 0.05). (D) The Manhattan plot shows the taxonomic information of OTUs enriched (*p* < 0.05, log-fold change >1) in the 1% straw addition treatment compared to the 0 and 3% straw addition treatment. The filled circles represent significantly enriched OTUs, while the open circles indicate OTUs with no significant difference. The dashed line represented the threshold of significance (Benjamini–Hochberg-corrected *p* = 0.05). 0, 1, and 3% represent the proportions of straw added to 200 g of soil, with no addition, 2 g, and 6 g of straw, respectively; OTU, operational taxonomic units.

Rarefaction curves of OTUs at 97% sequence similarity of all samples tended to approach the saturation plateau (Supplementary Figure S3). Therefore, the sequencing depth was adequate to assess the fungal diversity of our samples. With 1 and 0% treatments, fungal alpha diversity indices (Shannon and Inverse Simpson indices) in tomato rhizosphere were significantly higher than the 3% treatment (Tukey’s HSD test, *p* < 0.05; Figure 3A). However, no significant difference was observed between 1 and 0% treatments. Based on the Bray–Curtis dissimilarities, PCoA revealed that soil samples from the same treatment grouped together, while three treatments were separated from each other (Figure 3B).

Our study found that straw addition played a significant role in inducing several fungal differential OTUs among three treatments. This observation was consistent across all samples analyzed, where 12 bacterial phyla were detected. Among them, Ascomycota, Basidiomycota, and Mortierellomycota were the dominant phyla (average relative abundances>1%) in at least one treatment. It is worth noting that fungal community composition did not uniformly respond to different amounts of straw addition (Supplementary Figure S4).

We found that straw addition induced several fungal differential OTUs. The likelihood ratio test found that 46 were enriched and 31 OTUs were depleted in the 1% straw addition treatment compared to the 0%, respectively. In contrast, 21 OTUs were enriched and 61 OTUs were depleted in the 1% straw addition treatment compared to the 3% straw addition, respectively (Figure 2C). These stimulated OTUs mainly belonged to Ascomycota and Basidiomycota. In particular, 1% straw addition stimulated the OTU86 (*Solicoccozyma* sp.), OTU120 (*Schizothecium* sp.), OTU341 (*Coprinellus* sp.), OTU544 (*Schizothecium* sp.), OTU620 (*Preussia* sp.), compared to the 0% straw addition. Compared to the 3% treatment, 1% straw addition also stimulated the OTU7 (*Acaulium* sp.), OTU104 (*Acaulium* sp.), and OTU470 (*Remersonia* sp.) (Figure 3D).

### 3.4 Correlation analysis between plant growth and microbial community

**Figure 4:**
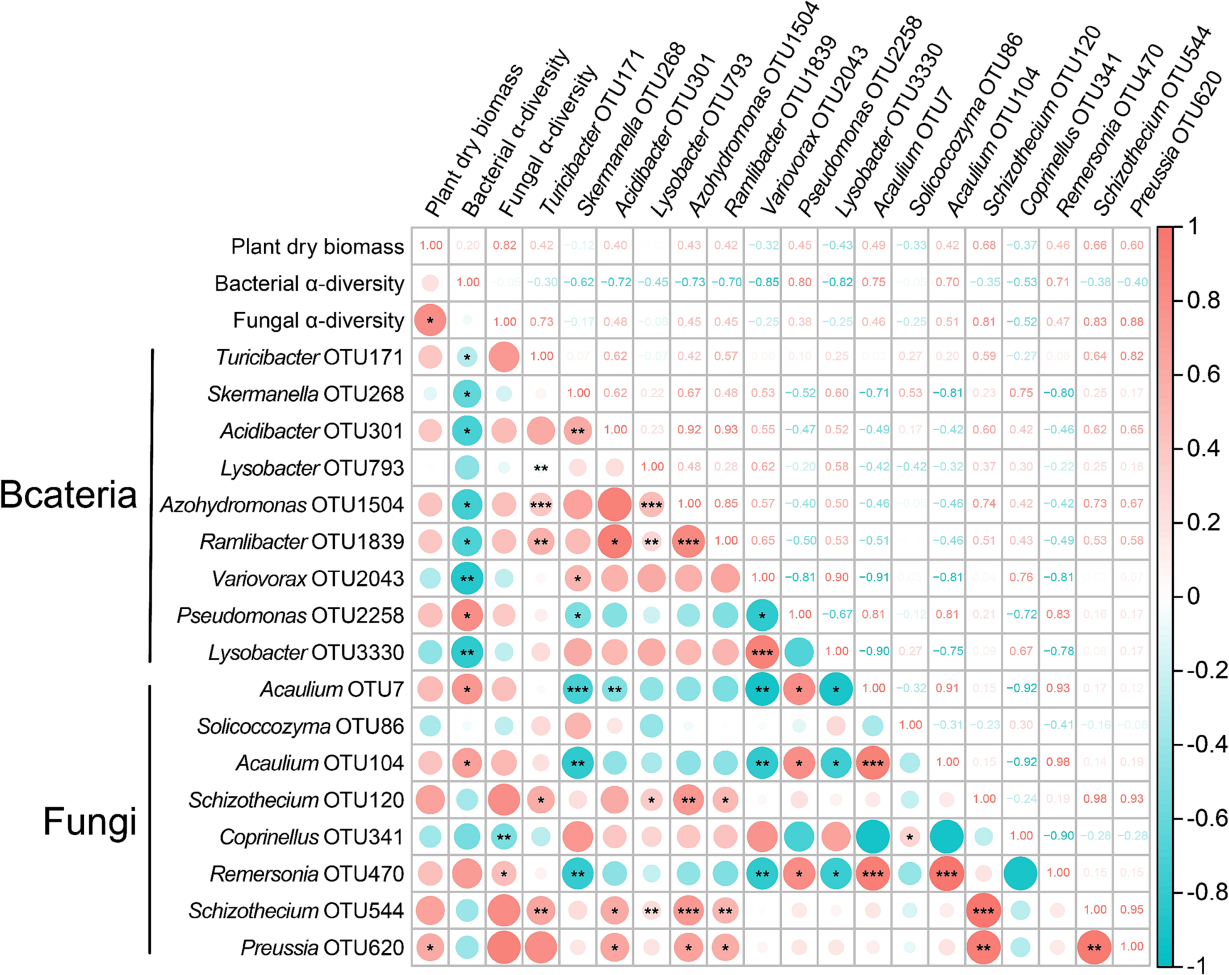
The heatmap matrix of Spearman’s correlation described the prospective relationship between plant growth and microbial community. Pink represents a positive correlation, and blue represents a negative correlation. The darker the color, the stronger the correlation, and vice versa. The numbers in the boxes represent the correlation coefficients. Significance levels are as follows: *: *p* < 0.05, **: *p* < 0.01, ***: *p* < 0.001.

Based on the preliminary analysis of the changes in microbial community structure and function, in order to further clarify whether these changes affect the growth of tomato seedlings, we conducted a correlation matrix using Spearman (Figure 4). The results demonstrated that both fungal *α*-diversity and OTU620 (Ascomycota) showed significant positive correlations with tomato biomass accumulation (*R*^2^ = 0.82, *p* < 0.05; *R*^2^ = 0.60, *p* < 0.05). This suggests that the changes in fungal community OTUs stimulated by the addition of Ricinus straws may play a crucial positive regulatory role in tomato growth.

### 3.5 The Ricinus straw-induced changes of *Fusarium* sp.

**Figure 5:**
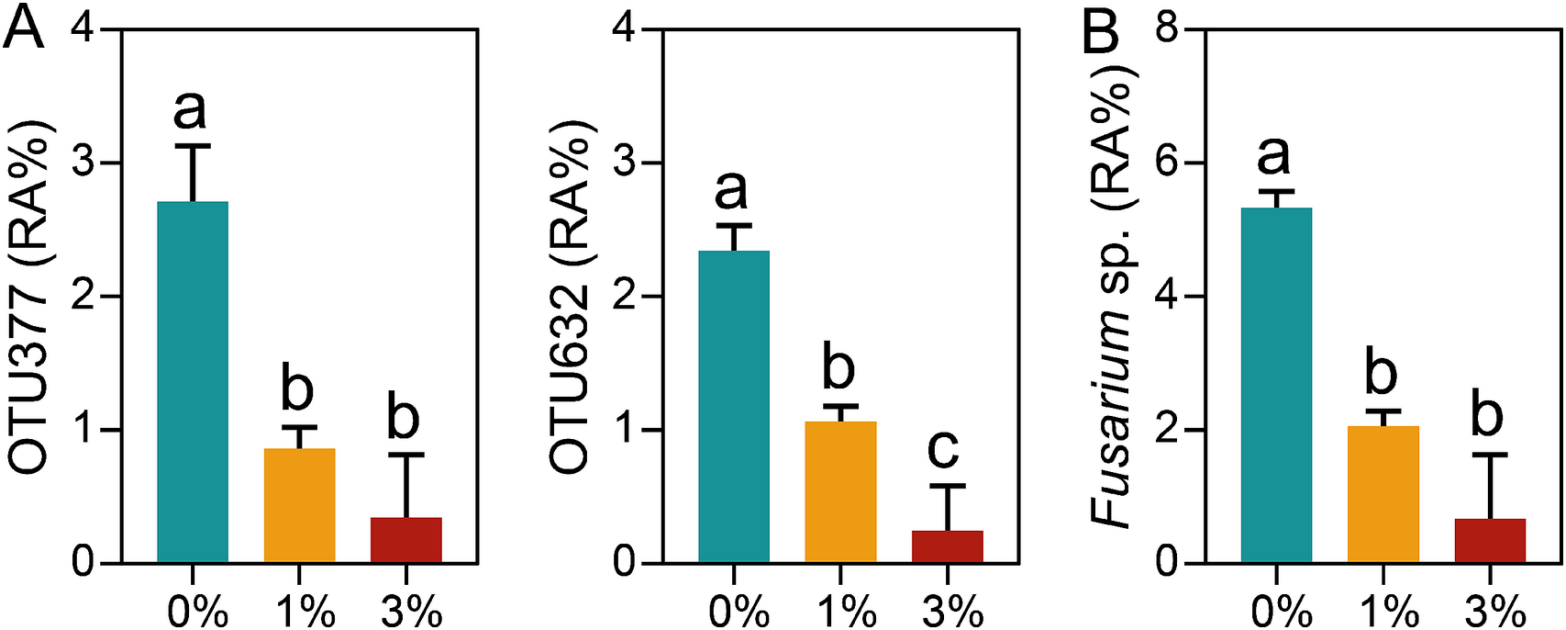
Changes of *Fusarium* sp. induced by Ricinus straw. (A) Analysis of the abundance of differential OTUs (*Fusarium* sp.) with average relatively high relative abundance of >0.5% in at least one treatment. (B) The relative abundance of *Fusarium* sp. The data are shown as mean ± SE (*n* = 3). Different letters indicate significant differences (Tukey’s HSD test, *p* < 0.05).

In this study, we also found that the addition of Ricinus straw could reduce the abundance of plant pathogens. Here, we focused particularly on the soilborne pathogen *Fusarium* sp. The results showed that the addition of Ricinus straw significantly reduced the relative abundance of OTU377 (*Fusarium* sp.) and OTU632 (*Fusarium* sp.). In addition, compared to the 0% treatment, the *Fusarium* sp. abundance in 1 and 3% Ricinus straw addition treatments was reduced by 61.40 and 87.28%, respectively (Figure 5).

### 3.6 Ricinus straw extract inhibited the growth of FOL

**Figure 6:**
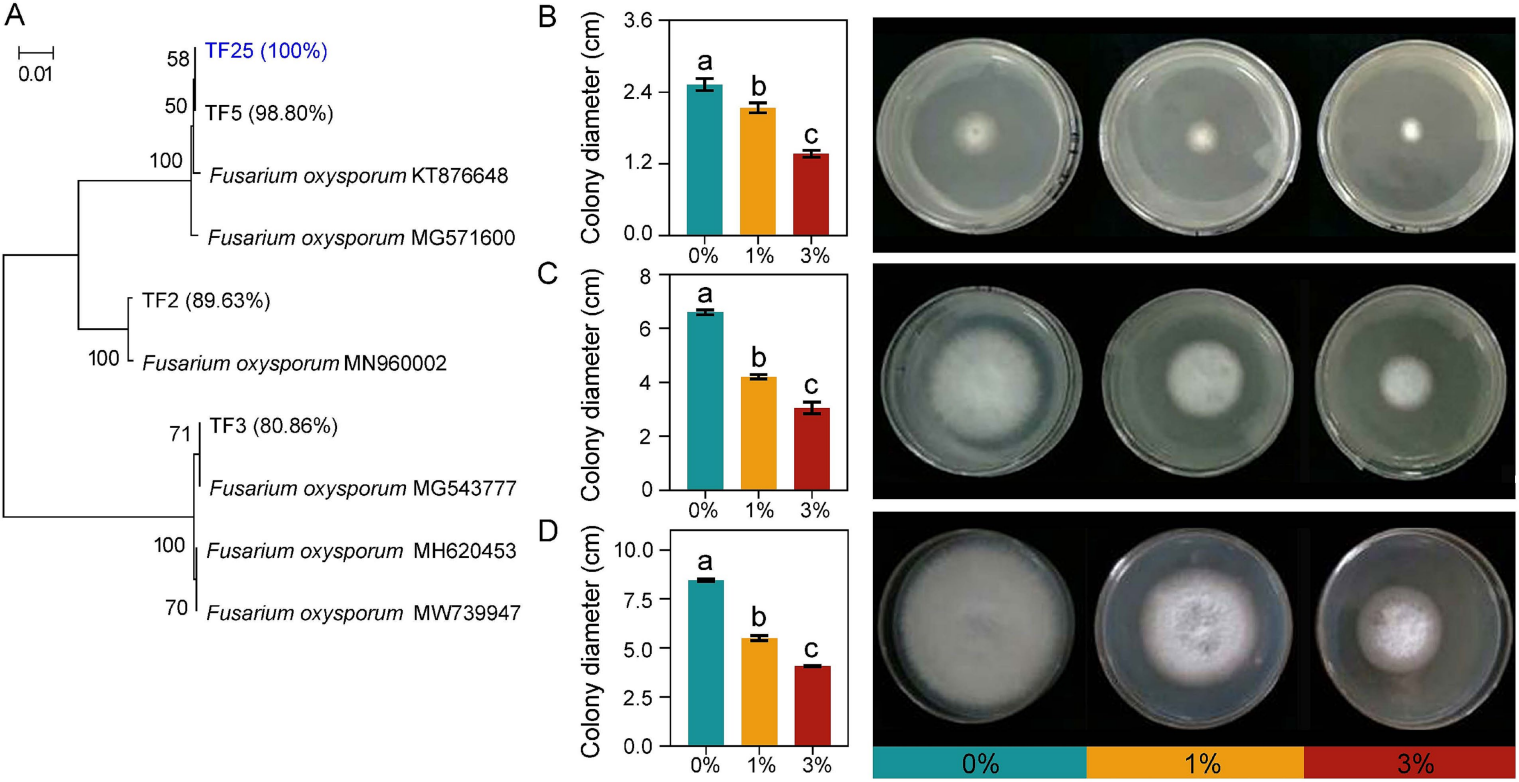
Isolated FOL and the impact of Ricinus straw extract on the growth of TF25. Neighbor-joining trees showing the phylogenetic relationships of isolated FOL. Numbers in parentheses are the sequence similarities of each FOL strain with OTU632. Bootstrap values are based on 1,000-fold resampling and are shown at the branching points (A). The photographs show the colony morphologies of TF25 grown on PDA. Effect of Ricinus straw extract on the growth of FOL: colony diameter (cm) in solid medium PDA at 2nd (B), 4th (C), and 6th (D) days of cultivation, respectively. Different letters indicate significant differences (Tukey’s HSD test, *p* < 0.05).

Through sequencing and alignment of the isolated strains, we obtained three strains of FOL. Based on this result, we selected one of the FOLs, named TF25, for further purification and preservation because OTU632 has a 100% sequencing similarity with it. Subsequently, we assessed the impact of Ricinus straw extract on the growth of TF25. The results showed that the castor bean straw extract significantly inhibited the colony diameter of TF25, and with the increase of the extract concentration, the inhibitory effect on the growth of TF25 became more obvious at the 2nd, 4th, and 6th days of cultivation. Particularly, on the 6th day of cultivation, compared to 0% treatment, the inhibition rates of 1 and 3% Ricinus straw extract on the growth of TF25 reached 34.95 and 51.91%, respectively (Figure 6). This discovery will aid our understanding of how straw addition can alleviate soilborne diseases in crops.

## 4 Discussion

Straws can increase the levels of the availability of soil nitrogen, phosphorus, and potassium. Additionally, the humic acids and fulvic acids produced during the decomposition process contribute to forming stable soil aggregates, creating suitable pore spaces, improving the environment for root growth, and supporting plant growth (Zhang et al., 2015; Acharya and Pesacreta, 2022; Cui et al., 2022). In this study, we found that in the later stage of straw decomposition, the 1% addition treatment promoted the accumulation of tomato dry matter (Figure 1B). However, during the mid-stage of straw decomposition, the growth of tomatoes was significantly inhibited, and the inhibitory effect was more pronounced with excess straw addition (Figure 1A). The majority of the nutrients in straws exist in organic form, with relatively few in mineral form. Chemical studies have shown that Ricinus straw tissues contain a variety of chemical components, including terpenoids, phenolic acids, alkaloids, and flavonoids, and the extracts of Ricinus straw possess a broad range of pharmacological activities (Raji et al., 2006; Wafa et al., 2014; Farooq et al., 2018; Sbihi et al., 2018). Organic decomposition is slow, and the short-term release of nutrients into the soil is not significant leading to soil C/N imbalance; excessive straw addition can increase the soil C/N ratio, and available soil N is immobilized to the point where plants must compete with the microbial community for available soil N pools (Zhao et al., 2016), resulting in immobilization of available soil N and inhibiting dry matter accumulation. Straw decomposition is a dynamic process, and due to the difficulty in improving soil nutrient conditions in a timely manner and the high C/N ratio of the straws, the formation of soil aggregates may need the involvement of microbes. Moreover, in the aggregation process, microbes can absorb and utilize some soil nutrients, negatively affecting crop growth (Li et al., 2021; Lv et al., 2024). On the other hand, the presence of polymers such as cellulose and lignin in straws slows down the efficiency of biological decomposition, thereby impeding root penetration and inhibiting crop growth, especially during the mid-stage of decomposition (Su et al., 2016).

As a crucial part of the soil ecosystem, bacteria play a significant role in organic matter decomposition (Wang et al., 2017), nutrient transformation, and nutrient cycling (Marschner et al., 2011; Ijaz et al., 2023). Bacteria, particularly Actinobacteria and Firmicutes, tend to decompose unstable organic compounds and dominate in the early stages of straw decomposition (Paterson et al., 2011). Our results showed that straw addition did not affect the *α*-diversity of the bacterial community (Abulaizi et al., 2023), but altered the abundance of the community (Figure 2). Some studies have shown that during the decomposition of straw, the Actinobacteria and Firmicutes exhibit similar dynamic changes with the rate of CO_2_ release, indicating that they play a crucial role in the decomposition process compared to other bacterial species (Zhao et al., 2016). However, our results suggested that the Actinobacteria was decreased by the addition of 1% straw treatment. The possible reason for this is that the addition of an appropriate amount of straw gradually resulted in the fungal becoming dominant in the later stages of the decomposition process, thereby reducing the abundance of bacterial communities associated with straw decomposition. Meanwhile, positive plant–microbe interactions derive benefit from the increase in the abundance of beneficial microbes associated with plants. Our high-throughput amplicon sequencing analysis showed that the addition of 1% Ricinus straws increased the relative abundance of bacterial taxa in the tomato rhizosphere that have the potential to promote plant growth. For instance, Proteobacteria had a higher abundance in the bacterial community of tomato rhizosphere soil in the late stage of straw decomposition compared to the 0% treatment. The enrichment effects also existed with different amounts of Ricinus straws, and the 1% additive amount elevated the relative abundance of Acidobacteria than that of the 3% additive amount. In natural environments, Acidobacteria plays a crucial role in decomposing complex organic polymers, such as lignocellulose, starch, and proteins, found in crop straw (Liu et al., 2016). The appropriate amount of straw addition could induce an increase in the relative abundance of Acidobacteria, thereby providing beneficial nutrients for tomato growth. However, adding 3% Ricinus straw significantly increased the relative abundance of Firmicutes compared to other treatments.

Soil microorganisms can influence plant growth through symbiotic, mutualistic, or parasitic relationships. Adding Ricinus straws increased the relative abundance of some plant-beneficial microorganisms, such as *Ramlibacter* sp., an effective bacterium in decomposing aromatic compounds (Sun et al., 2019). Through processes such as adsorption and transport, *Ramlibacter* sp. can effectively break down aromatic compounds into substances beneficial for tomato growth, thereby preventing environmental pollution and inhibiting crop growth. Compared to the 0 and 3% treatments, the addition of 1% Ricinus straws significantly enriched the nitrogen-fixing bacterium *Azohydromonas* sp. in the rhizosphere of tomatoes (Gage, 2004). This bacterium tends to utilize soil nitrogen sources to participate in rhizospheric nitrogen cycling and show sensitivity to nitrogen. Therefore, adding an appropriate amount of straw can promote tomato growth by stimulating changes in the structure of dominant nitrogen-fixing bacterial communities. In addition, studies have shown that *Skermanella* sp., *Lysobacter* sp., Var*iovorax* sp., and *Pseudomonas* sp. play crucial roles in soil nitrogen fixation (Hu et al., 2021; Wang et al., 2023), antagonism against pathogenic bacteria (Qiao et al., 2024), and regulation of plant growth (Zhou et al., 2024).

The (hemi-) cellulosic polysaccharides and aromatic polymer lignin attached to the plant cell wall are essential components of straw (Kang et al., 2019). In this regard, some microorganisms with specific decomposition functions have been widely studied (Rastogi et al., 2010). It is generally believed that microbial communities can produce various cellulases, and fungi are the main producers of lignocellulose enzymes. They can decompose polysaccharides in straw into monosaccharides, which are then used in various fungal metabolic pathways and withstand the drastic changes in the nutritional environment during the straw decomposition process (Liu et al., 2021). This also indicates that fungi are more inclined to decompose more complex organic compounds and dominate in the later stages of straw decomposition (Marschner et al., 2011). In our study, we found that the addition of Ricinus straws altered the community composition of rhizosphere fungi in the tomato, especially the 1% additive amount showing more significant effects (Figure 3). Our high-throughput amplicon sequencing analysis showed that the dominant fungal phyla were found to be Ascomycota, Basidiomycota, and Mortierellomycota. The high abundance of Ascomycota may be due to their easy colonization and rapid growth characteristics. However, the addition of straw significantly increased the relative abundance of Basidiomycota, which shows slower growth (Kuramae et al., 2013; Liu et al., 2017). Studies have found that Basidiomycota can decompose and mineralize lignin, a type of aromatic polymer that is mainly resistant to alteration by other microorganisms (Cragg et al., 2015). Therefore, adding an appropriate amount of Ricinus straws can enhance the decomposition rate of straw by enriching the key decomposing microbial community (Basidiomycota), thereby providing necessary nutrients for tomato growth.

It is worth noting that putative beneficial microbial communities, such as *Schizothecium* sp., can significantly influence the overall functioning of the decomposer complex. *Schizothecium* sp. is particularly noteworthy for its ability to colonize the roots of various plants, forming a symbiotic complex between the fungus and the roots. This symbiotic relationship is particularly important, as it allows *Schizothecium* sp. to secrete various extracellular hydrolytic enzymes, such as cellulases, amylases, and laccases, which play a crucial role in promoting host plant growth and enhancing crop resilience to environmental stress (Usuki and Narisawa, 2007; Andrade-Linares et al., 2011). Additionally, *Solicoccozyma* sp., *Coprinellus* sp., and *Remersonia* sp. play important roles in promoting seed germination and plant growth and inhibiting the growth of pathogenic microorganisms (Carvajal et al., 2024; Ran et al., 2024; Xiao et al., 2024). In our study, the addition of Ricinus straws led to a significant increase in the relative abundance of the tomato rhizosphere fungal *Schizothecium* sp. and *Acaulium* sp. The cellulose-decomposing fungi efficiently promote the conversion of lignin and cellulose into humus, thereby positively regulating tomato growth (Figure 4).

The rhizosphere microbiome, as the second genome of plants, plays a crucial role in plant growth, development, and health (Berendsen et al., 2012). This study further assessed the functional significance of rhizosphere microbiota, demonstrating that the Ricinus straw-induced alterations in the tomato rhizosphere microbial community are important factors in promoting tomato growth. Furthermore, this study also demonstrated that identifying key microbial groups in crop rhizospheres is significantly important for enhancing agricultural sustainability, offering a promising avenue for future research. However, this study was conducted under microcosm conditions, and further field applications are needed to assess the actual growth-promoting effects of Ricinus straw on the tomato.

Ricinus straw, as a medicinal plant, has been extensively studied for the medicinal value of its leaves, stems, and roots, and has been used in the medical field for the treatment of skin cancer. In addition, extracts from Ricinus straw leaves exhibit resistance against various pathogenic bacteria such as *Candida albicans*, *S. aureus*, and *P. aeruginosa* (Suurbaar et al., 2017). This study also confirmed the antibacterial effects of Ricinus straw stalk extract *in vitro* and demonstrated a concentration-dependent inhibition of FOL growth. On the other hand, Ricinus straw stalks also reduced the relative abundance of the putative root-pathogenic microorganism *Fusarium* sp. in the tomato rhizosphere. Therefore, we thought that the degradation process of Ricinus straw stalks could release metabolites with antibacterial activity. These metabolites induced microbial community changes and inhibited the growth of pathogens, benefiting plant growth (Jin et al., 2024).

## 5 Conclusion

Overall, Ricinus straw has proven to be a game changer in agriculture, and the effect of straw on plants was highly dependent on the length of the straw decomposition. During the mid-stage of straw decomposition, it can have adverse effects on plant growth. However, in the later stages of decomposition, adding 1% of Ricinus straw, rather than a large amount, can significantly improve the growth of tomato plants induced by the changes in the soil microbial community. The straw addition significantly influenced the abundance, diversities, and composition of soil microbial communities, particularly by enhancing the relative abundance of potentially beneficial bacteria and fungi that play crucial roles in the decomposition of straw and the promotion of plant growth. These include genera such as *Ramlibacter* sp., *Azohydromonas* sp., *Schizothecium* sp., and *Acaulium* sp. In addition, it also contributed to the reduction in the relative abundance of *Fusarium* sp. These advancements have not only deepened our understanding of the functions of microbial communities induced by Ricinus straw addition but also opened up new possibilities for sustainable development in agriculture.

## Data Availability

The data presented in the study are deposited in the National Center for Biotechnology Information repository, with accession numbers PRINA1138190 and PRINA1138193.
