# Supplementary material for: The impact of Ricinus straw on tomato growth and soil microbial community
Source: Front Microbiol. 2024 Dec 2;15:1499302. doi: 10.3389/fmicb.2024.1499302 (PMC11646993; doi:10.3389/fmicb.2024.1499302)
Supplement: Supplementary file 1 [file Data_Sheet_1.docx]

# Supplementary Materials


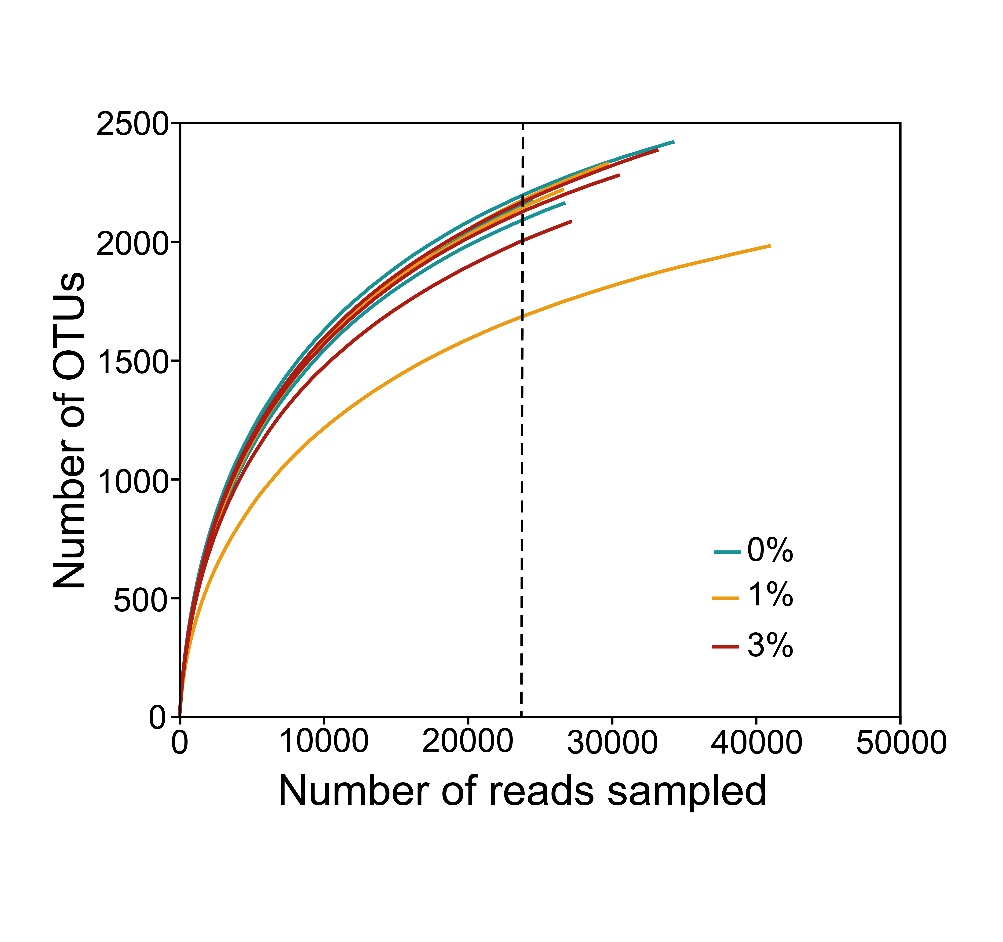


FIGURE S1. Rarefaction curves of the number of OTUs at the 97% sequence similarity. The dashed black line indicates the selected rarefaction depth (23796 sequences per sample) used to calculate the alpha diversity of the bacterial community in tomato seedling rhizosphere.


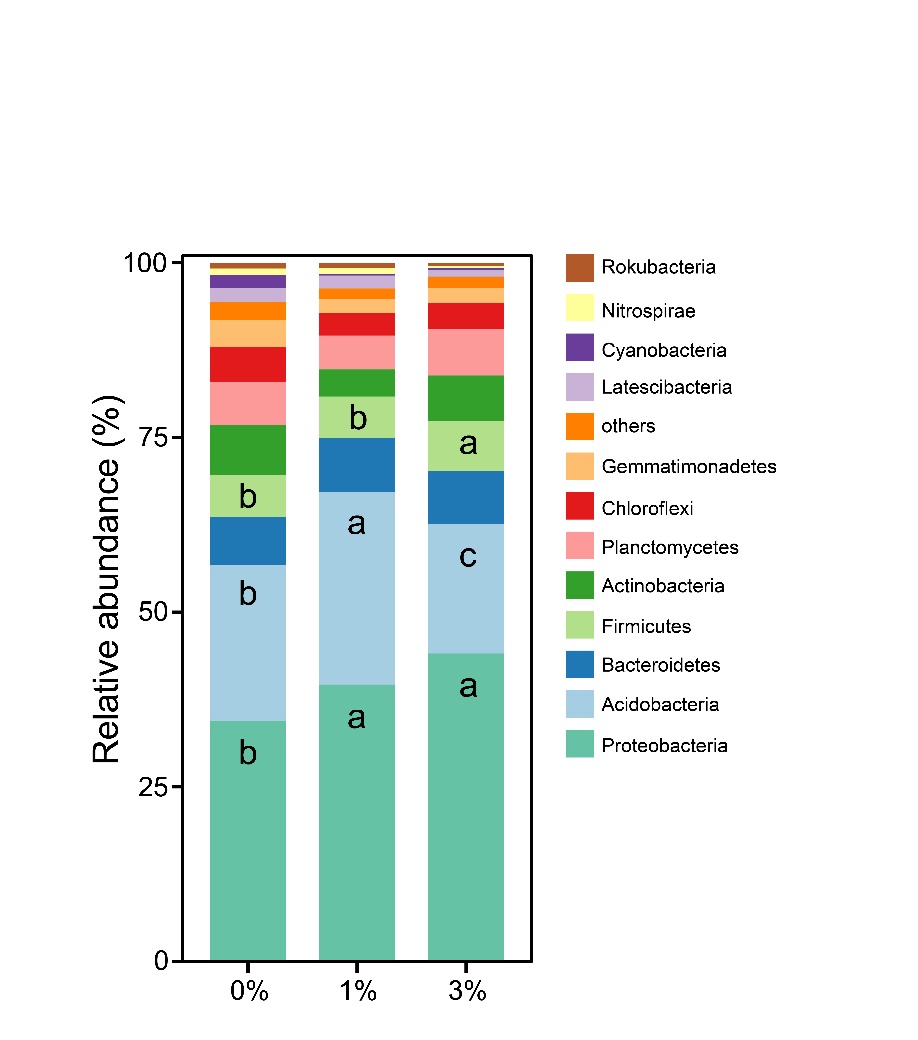


FIGURE S2. Relative abundances of main bacterial phyla (mean relative abundances > 0.5%) of each treatment. Each bar represents the mean value of three replicates. Data are shown as mean ± SE (n=3). Different letters indicate significant differences (Tukey's HSD test, *p* < 0.05).


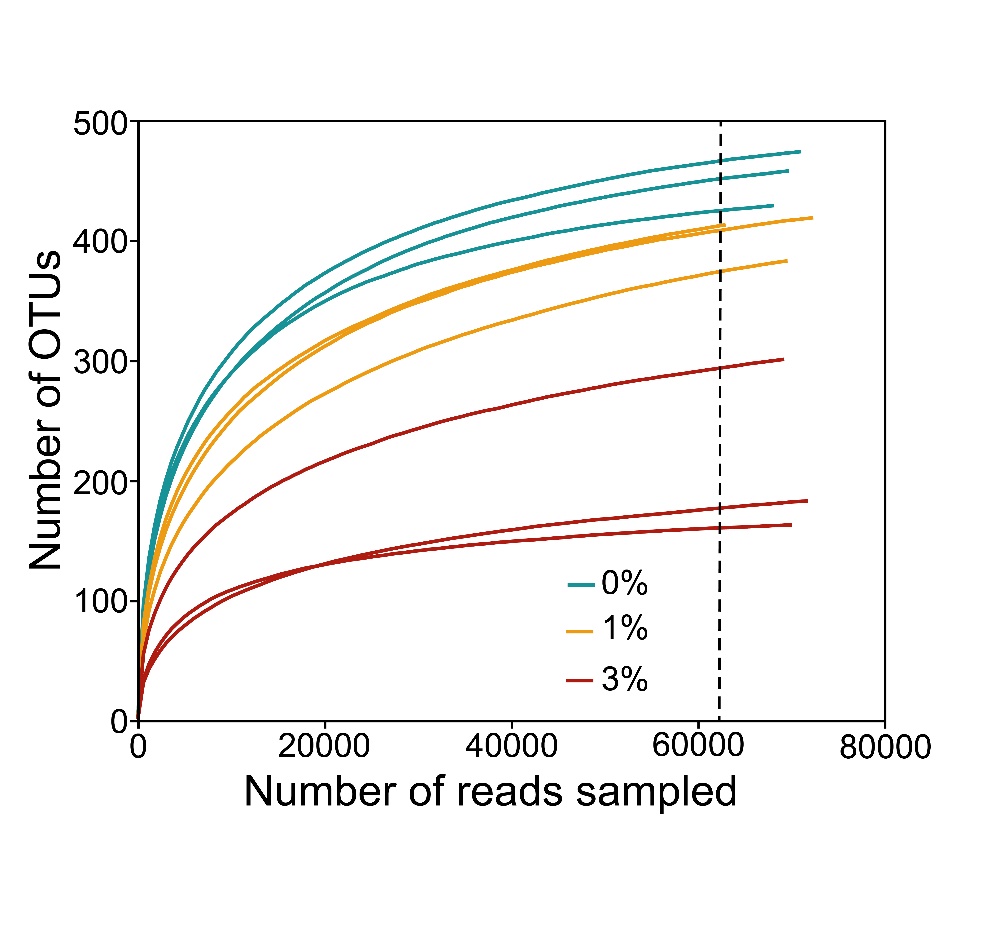


FIGURE S3. Rarefaction curves of the number of OTUs at the 97% sequence similarity. The dashed black line indicates the selected rarefaction depth (62168 sequences per sample) used to calculate the alpha diversity of the fungal community in tomato seedling rhizosphere.


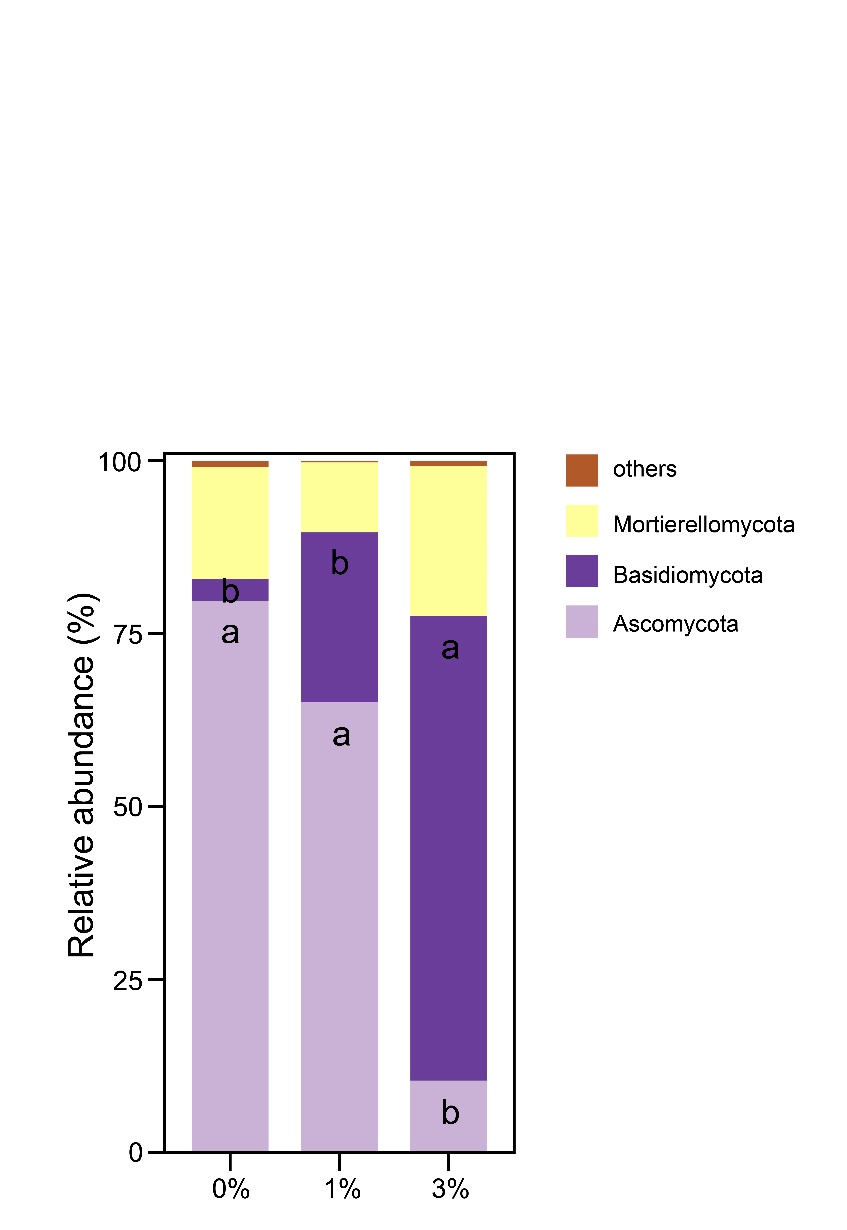


FIGURE S4. Relative abundances of main fungal phyla (mean relative abundances > 1%) of each treatment. Each bar represents the mean value of three replicates. Data are shown as mean ± SE (n=3). Different letters indicate significant differences (Tukey's HSD test, *p* < 0.05).
